# *P53* status, and G2/M cell cycle arrest, are determining factors in cell-death induction mediated by ELF-EMF in glioblastoma

**DOI:** 10.1038/s41598-023-38021-z

**Published:** 2023-07-05

**Authors:** Romina Mehdizadeh, Alireza Madjid Ansari, Flora Forouzesh, Fatemeh Shahriari, Seyed Peyman Shariatpanahi, Ali Salaritabar, Mohammad Amin Javidi

**Affiliations:** 1 Department of Integrative Oncology, Breast Cancer Research Center, Motamed Cancer Institute, ACECR, Tehran, Iran; 2 Department of Genetics, Faculty of Advanced Science and Technology, Tehran Medical Sciences, Islamic Azad University, Tehran, Iran; 3 Institute of Biochemistry and Biophysics, University of Tehran, Tehran, Iran; 4 Department of Molecular Genetics, Faculty of Biological Sciences, Tarbiat Modares University, Tehran, Iran

**Keywords:** Biophysics, Cancer, Cell biology, Genetics, Oncology

## Abstract

The average survival of patients with glioblastoma is 12–15 months. Therefore, finding a new treatment method is important, especially in cases that show resistance to treatment. Extremely low-frequency electromagnetic fields (ELF-EMF) have characteristics and capabilities that can be proposed as a new cancer treatment method with low side effects. This research examines the antitumor effect of ELF-EMF on U87 and U251 glioblastoma cell lines. Flowcytometry determined the viability/apoptosis and distribution of cells in different phases of the cell cycle. The size of cells was assessed by TEM. Important cell cycle regulation genes mRNA expression levels were investigated by real-time PCR. ELF-EMF induced apoptosis in U87cells much more than U251 (15% against 2.43%) and increased G2/M cell population in U87 (2.56%, *p value* < 0.05), and S phase in U251 (2.4%) (data are normalized to their sham exposure). The size of U87 cells increased significantly after ELF-EMF exposure (overexpressing *P53* in U251 cells increased the apoptosis induction by ELF-EMF). The expression level of *P53*, *P21*, and *MDM2* increased and *CCNB1* decreased in U87. Among the studied genes, *MCM6* expression decreased in U251. Increasing expression of *P53*, *P21* and decreasing *CCNB1*, induction of cell G2/M cycle arrest, and consequently increase in the cell size can be suggested as one of the main mechanisms of apoptosis induction by ELF-EMF; furthermore, our results demonstrate the possible footprint of *P53* in the apoptosis induction by ELF-EMF, as U87 carry the wild type of *P53* and U251 has the mutated form of this gene.

## Introduction

Brain tumors are among the most fatal form of cancers^1,2^. There are different categories applied to these tumors according to the histological characteristics, cellular origin (primary brain tumors and secondary brain tumors ^3^), shape, and related tissue^4^. WHO divides brain tumors into two main categories; benign tumors and malignant tumors. Benign tumors which may called low-grade (grade I and II) are non-cancerous, non-aggressive, and have a slow progression rate; whereas, malignant tumors are considered as high-grade (grade III and IV), either the primary ones (originated from the brain tissue) or secondary ones (spread to the brain from elsewhere due to the metastasis) are aggressive, have a rapid progression rate, and tend to metastasis to other organs in the body ^1^. According to the WHO classification with the concept of histological features, the majority of primary brain tumors belong to the neuroepithelial, such as Gliomas^5^. Gliomas are the most common malignant tumors of the central nervous system (CNS) that arise from the glial cells. Glioblastoma multiforme (GBM) belongs to the grade IV gliomas and is the most aggressive type of brain tumor. Unfortunately, the GBM is one of the most poor-prognosis cancers, even with the advances and aid of the treatments approaches like surgery, chemotherapy, and radiation therapy^6^.

Electromagnetic fields (EMFs) are a group of radiations that can be produced by electrical currents in human-made devices or natural sources. EMFs are divided into two main groups: non-ionizing and ionizing. EMFs are also categorized based on their frequency or wavelength (the radiation energy is directly proportional to the frequency; in the non-ionizing part, the radiation energy is too weak to break chemical bonds, on the other hand, ionizing fields have high energy and can break chemical bonds ^7^).

Amounting of studies have reported the anti-cancer effects of the extremely low-frequency EMFs (ELF-EMFs) (0–300 Hz) against various cancers. Many pieces of research performed till now trying to shed light on the underlying molecular mechanism of action of these fields; exposure time, frequency, intensity, and cell type seem to be some important factors in exerting this anti-cancer effect; however, the exact mechanism seems no to be sufficiently transparent yet^8^. One of the main mechanisms which are proposed to the sibling of these fields (electrical fields, renowned as tumor treating field (TTF), which has FDA approval for use in recurrent or early diagnosed GBM patients) is through hindering the mitotic microtubules fibers, inducing the cell cycle arrest in the metaphase and regarding cell to the apoptosis ^6^.

Magnetic fields, have some benefits over electrical fields, including higher permeability; this is more crucial when we realize that, the obstructing physical properties including the higher resistance are high in the organs like the head (about 50 Ω in the scalp, skull, and gray matter together ^9^. The main aim of this study was to investigate the anti-tumor effect and plausible cellular/molecular mechanism of action of ELF-EMF on two human glioblastoma cell lines U87 and U251 with distinct genetic patterns, specifically according to their *P53* status (U87 has the wild type of this gatekeeper gene, but U251 has mutated form^10^).

## Materials and methods

### Cell culture

Human U87 and U251 MG glioblastoma cell lines and normal human fibroblast (purchased from Pasteur institute of Iran) were cultured in a 25 cm^2^ flask (Nunc, Thermo Fisher) in h-DMEM (High-glucose Dulbecco's modified Eagle’s medium) with 10% FBS, 1% penicillin and streptomycin (all from Gibco, Thermo Fisher, USA) at 37 °C in a humidified atmosphere of 5% CO_2_ in the incubator (Memmert, Germany). The medium was replaced with a fresh complete medium every 4 days. When cells reached around 80% confluence, they were trypsinized, counted, and then seeded in each well of 6-well plates with a density of 5 × 10^4^. We further extended the experiments on the MCF-7 cell line to assess how wild-type *P53* status in these cells may be effective in the apoptosis induced by ELF-EMF (the culturing and exposure conditions of these cells were the same as the previous ones).

### ELF exposure characteristics

**Figure 1:**
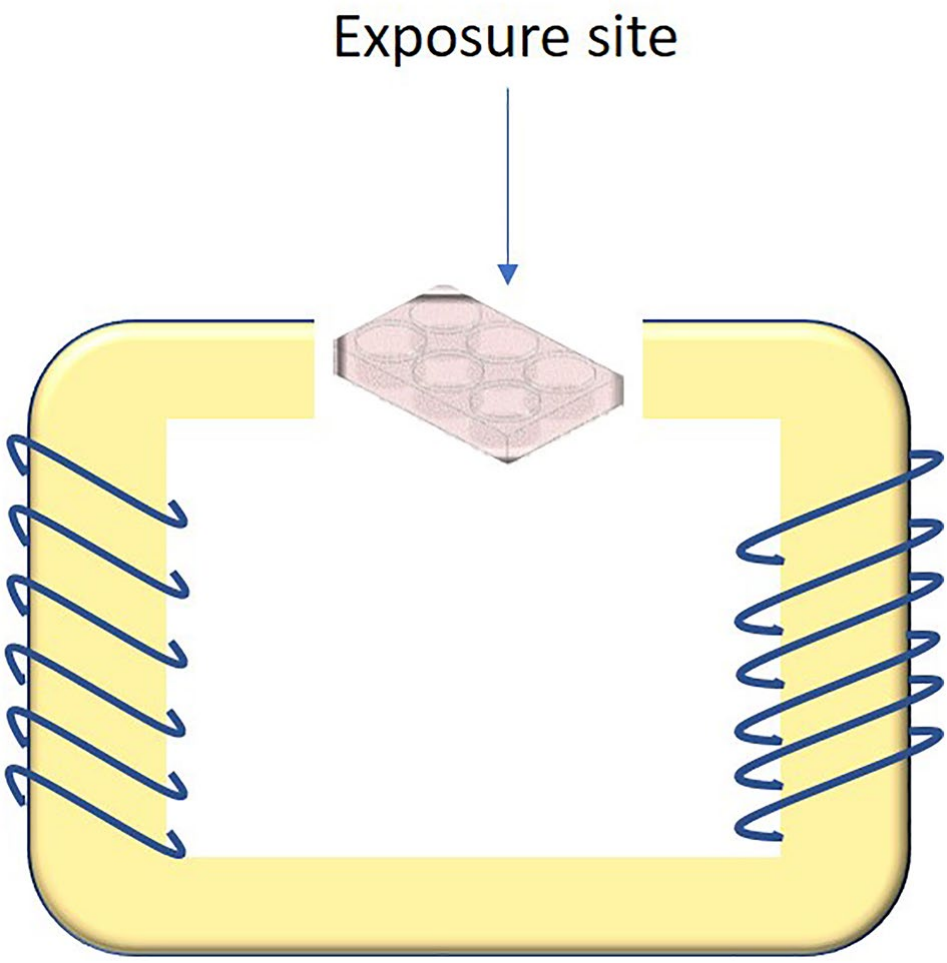
ELF-EMF schematic view and the exposure site.

The ELF-EMF exposure device (Parto Farazane Farda, Iran) consists of two pairs of square coils which were manufactured in a U-shape solenoid-like configuration, with an iron core to ensure maximal homogeneity and arise levels of B-field. The coils were wrapped with 1250 single-strand copper wires ^11^. The exposure site dimensions are a 15 × 15 × 15 cm box. A waveform generator (GWInstek SFG-1000 series, South Korea) was linked to the coils for obtaining a magnetic field that could be calibrated between 1–100 mT and 1 to 60 Hz respectively ^12^ (as described before ^11^) (Fig. 1). U87, U251, and fibroblast cells were exposed to ELF-EMF with the frequency of 1 Hz, and 100 mT intensity, for 5 days, 2 h each day. Sham exposure as the control of this study had the same condition as the exposed groups but the generator set is off. So, the field condition at the sham exposed group was equal to the background level (around 110 µT). All of the area related to the exposure site has been thoroughly calibrated by using the Lakeshore® Gauss meter on a net-like plastic plate situated at bottom of the exposure place. Following calibration, an 8 × 8 cm square at the middle of the exposure site, in which the field fluctuation is less than 2%, was considered an exposure area. Also, a cold air conditioner has been situated near the iron core and coils to reduce probable overheat. Although the temperature setting would not exceed the maximum 37º C based on the thermodynamic predictions, the cold air current would guarantee the temperature stabilizing during the field generation.

### Cell proliferation assay

All three cell lines were seeded, at a density of 0.5 × 10^5^ cells/ml in DMEM medium. after 5 days, exposed and shame exposed groups cells were harvested, washed, and re-suspended in 0.4% trypan blue in PBS solution. The number of viable cells was counted using Neubauer lam according to standard protocol (the cells which stained blue was considered dead cells and those that didn’t stain were considered live cells).

### Overexpressing P53

To assess the *P53* role in apoptosis induction by ELF-EMF, pCMV-Neo-Bam-H1-p53 plasmid was used. This plasmid was kindly provided by Dr. Michael Resnick (NIEHS, NIH), by the aid of BamHI restriction enzyme and cloning, cDNA of *P53* was inserted into the pCMV-NeoBam vector from Addgene (Cambridge, Massachusetts, USA) (reference^13^ shows the vector map). U251 cells were transfected with 100 ng/μl concentration of this plasmid (abbreviated as U251-P53), by Lipofectamine®2000.

### Flow cytometry

After 5 days of ELF exposure, U87 and U251 MG glioblastoma and fibroblast exposed and sham exposure both were collected by trypsinizing and centrifugation at 1200 rpm (Sigma, USA) to reach cell pellets. The pellets were then re-suspended in PBS (phosphate buffered saline, Gibco, USA), and utilized as the template for the flow cytometry test (BD FACSCalibur™). To assess the kind of cell death Annexin V/PI, (eBiosciencesDx) (the Annexin V-FITC IQ products, Netherlands) (20 and 5 μl, respectively) corresponding signal provides a very sensitive method for detecting cellular apoptosis, while propidium iodide (PI) (2 μl) (Sigma-Aldrich, Germany) is used to detect necrotic or late apoptotic cells, characterized by the loss of the integrity of the plasma and nuclear membranes) performed. We further investigated the cell cycle distribution of cells by determining the cellular DNA content with a saturating amount of DNA-binding dye. After 5 days of exposure to ELF-EMF, exposed and sham exposure cells were collected. These cells were washed twice with PBS and incubated with propidium iodide (10 μg/ml) and RNase A (0.1 mg/ml) (Sigma-Aldrich, Germany) for 30 min at 37 °C and then the distribution of cells in the different cell cycle phases was analyzed by Flow cytometer using FlowJo software v 10.2.

### Real-time PCR

Alteration in the mRNA expression level of *P21*, *P53*, *MDM2*, *MCM6*, and *CCNB1* in U87 and U251 cells after exposure was determined by using real-time PCR. Total RNA was extracted by using the TRIZol reagent (Invitrogen). Reverse transcription on the extracted RNAs was performed by using the Revert Aid First Strand cDNA Synthesis Kit (Thermo Fisher). These cDNAs were subsequently used as the template for real-time PCR. Primers were designed by using NCBI primer design software (Table1) (*Gapdh* used as housekeeping/internal control) to specifically amplify cDNAs of the mentioned genes. The real-time PCR reaction was performed on StepOnePlus™ real-time PCR device (Applied Biosystems, USA) in 96-well microtiter plates by using the SYBR Green PCR Master Mix (AMPLIQON, Denmark). The thermal cycling of stages consisted of an initial denaturation step at 95 °C for 15 min, 40 cycles at 95 °C for the 20 s, and 60 °C for 1 min, followed by a melt curve step.

**Table 1:** Primes sequence used for real-time PCR.

Gene name	Accession number	Primers Sequence (5' to 3')	Amplicon length(bp)
*P53*	NM-000546	F: TGTGACTTGCACGTACTCCC R: ACCATCGCTATCTGAGCAGC	199
*P21*	NM-000389	F: GACCATGTGGACCTGTCACT R: CGTTTGGAGTGGTAGAAATCTGTC	140
*MDM2*	NM-001145337	F: TCTGTGAGTGAGAACAGGTGTCR: TGGCGTTTTCTTTGTCGTTCA	191
*MCM6*	NM-005915	F: CAGCATTAAAGAGGAGCGAGC R: GCTCTCACTTCCCTCTGTGG	191
*CCNB1*	NM-001354844	F: GCACTTCCTTCGGAGAGCAT R: TGTTCTTGACAGTCCATTCACCA	188

### Transition electron microscopy (TEM)

After 5 days of treatment by ELF-EMF, U87, U251, and fibroblast cells were collected by trypsinizing and centrifuged at 1200 rpm. Transmission electron micrograph (TEM) images were recorded from the Pasteur Institute of Iran. For TEM analysis, the sample particles were dispersed in ethanol and then dropped onto copper grids with porous carbon films.

### Statistical analysis

The obtained data were evaluated by GraphPad prism8. The total of the study was measured as mean ± standard deviation (SD). For comparison between the two groups the t-test was applied, in cases where another statistic test has been applied, it is mentioned accordingly. Statistical significance was expressed as a *p-value* ˂ 0.05.

## Results

### Cellular morphology changes

#### Optical microscope analysis

**Figure 2:**
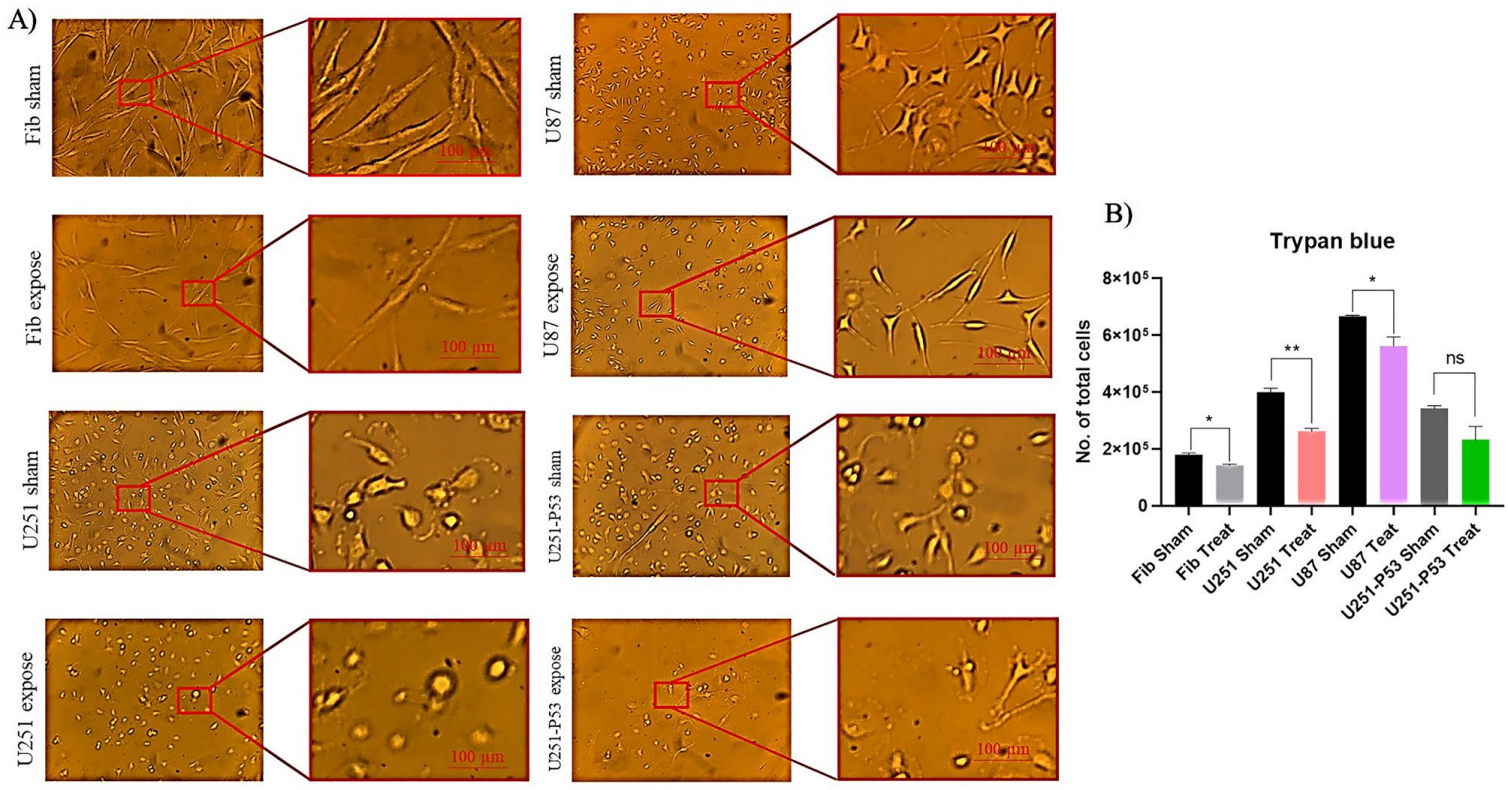
Cellular morphology and proliferation alteration under light microscope before and after ELF-EMF exposure. (**A**) After five days, the shape of cancerous cells which were exposed to the ELF-EMF altered and the rate of rounded cells (which may be interpreted as cells undergo apoptosis) was much more in them (fifth day, exposed U87 and U251); these shapes were rare in the fibroblasts. Comparing the two cancerous cells, the rate of rounded cells was much more in U87, furthermore, it seemed that cancerous cells weren’t able to proliferate as much as their sham exposure group. (**B**) Trypan blue results for the fifth day revealed that after exposure the total number of cells in the exposed groups is less compared with their corresponding sham exposure (the same number of cells were seeded for each exposed and sham exposure group (the first day: just before exposure)). This decrease was statistically significant in fibroblast, U251, and U87 cells (*: *p-value* < 0.05; **: *p-value* < 0.01); however, in the U251-P53 group this increase was not statistically significant (Mann–Whitney U test performed as statistical analysis to compare each exposed group with its sham exposure).

Morphological changes after exposure were determined in two groups (sham exposure and exposed) of all cells, firstly by an inverted optical microscope (Olympus). As shown in Fig. 2, after 2 h/day exposure to ELF-EMF, exposed U87, U251, and U251-P53 cell lines underwent morphological alteration concerning the shape and the size of the cells. The morphological analysis demonstrated that the shape of exposed U87, U251, and U251-P53 cell lines changed to a spherical shape on the fifth day. Whereas, in normal fibroblast cells there were no significant differences in exposed and sham exposure. Moreover, a reduction in the cell population on the fifth day was observed in exposed U87 cells. The trypan blue results demonstrated that after exposure the rate of proliferation decreased significantly in all cells after the fifth day of exposure compared with the unexposed cells (the sham exposure); although this decrease was not statistically significant in the U251-P53 group (*p value* = 0.0849) (Fig. 2).

### Annexin V/PI

**Figure 3:**
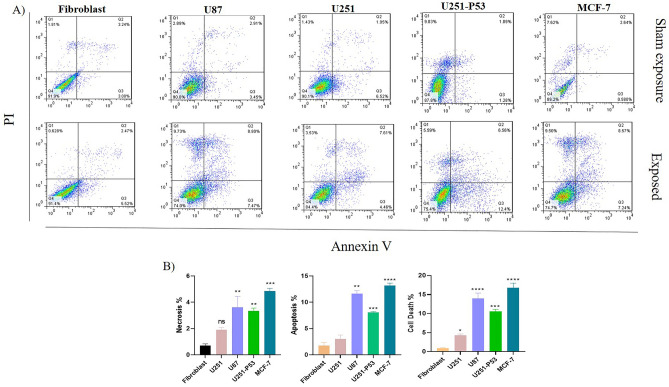
(**A**) Flow cytometry histogram showed the changes in the percentage rate of necrosis (Q1: PI+/AnnexinV−), late apoptosis (Q2: PI+/AnnexinV+), early apoptosis (Q3: PI−/AnnexinV+) and live cells (Q4: PI-/AnnexinV−). The percentage of live cells decreased significantly in U87, U251-P53, and MCF-7 compared to Fibroblast. (**B**) Flow cytometry diagram of 5 groups fibroblast, U251, U87, U251-P53, and MCF-7. This graph is normalized using sham exposure groups. Respectively, necrosis, apoptosis, and cell death rate in fibroblasts were calculated to be about 1.18%, 2.19%, and 0.95%, in U251 2.04%, 2.43%, and 4.55%, in U87 3.64%, 15%, and 11.28%, in U251-P53 3%, 8%, and 11.2%, and in MCF-7 5%, 13%, and 17.9%. According to the graphs, a significant increase in apoptosis and cell death was observed in the U87 U251-P53, and MCF-7 cell line compared to the fibroblast (*p-value < 0.05, **p-value < 0.01, ***p-value < 0.001, ****p-value < 0.0001) The experiments were performed twice for each cell line under the exposure. (The place of quadrants was normalized according to the sham exposure and the control group; the differences between exposed (exposed to ELF-EMF with 1 Hz, 100 mT, 2 h each day for 5 days) and sham exposure groups used to report the apoptosis, necrosis, and cell death; one-way ANOVA was applied).

Apoptosis assay was performed by the double staining method (Annexin V-FITC/PI kit) and the percentage of dead cells, early apoptosis, late apoptosis, and live cells were assessed by flow cytometry. Flow cytometry analysis showed that the percentage of apoptosis (early + late apoptotic cells), cell death, and necrosis were respectively in U87 (11.39%, 15.45%, and 3.64), U251 (2.43%, 4.55% and 2.04%), U251-P53 (8.15%, 10.7%, and 3.35%), MCF-7 (13.15%, 16.8%, and 4.85%), and Fibroblast (2.19%, 0.95% and − 1.18%). All the results were represented as the difference between the exposed groups and the corresponding sham exposure. Apoptosis rate increased significantly in U87 and U251 cell lines post-exposure; however, there were no differences in normal fibroblast. When comparing the two GBM cell lines, it was ascertained that ELF-EMF exposure could significantly increase the apoptosis rate in U87 compared to U251 (Fig. 3).

### Cell cycle

**Figure 4:**
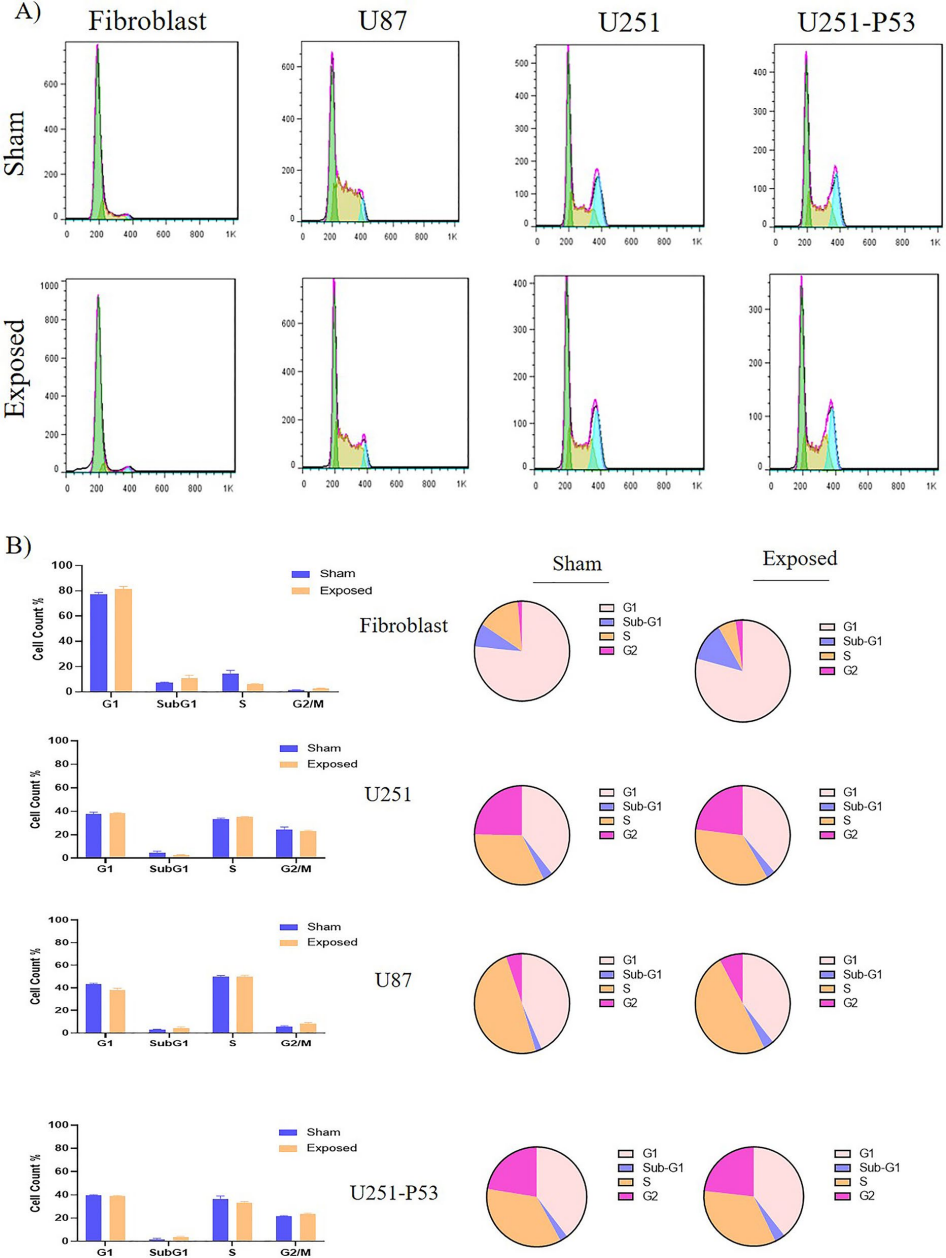
(**A**) Flow cytometry histogram showed alteration in the distribution of cells in different phases of the cell cycle in exposed (exposed to ELF-EMF with 1 Hz, 100 mT, 2 h each day for 5 days) and sham exposure groups of different cell lines. (**B**) Column (left) and pie chart (right) diagrams of flow cytometry results for the cell cycle. Respectively, subG1, G1, S, and G2/M phases distribution of cells in fibroblasts were 4.88%, 2.66%, − 8.43%, and 0.99%, in U251 were − 0.3%, − 1.1%, 2.4%, and − 1.79%, and (there was a slight increase in S phase), in U87 calculated to be 1.43%, − 4.6%, − 0.18%, and 2.56% (increased rate in G2/M phase), and in U251-P53 0.92, 0.28%, − 1.16%, and 1.1% (a slight increase in G1 and G2/M phases and slight decrease in S phase) (the minus results represent decreasing and the positive results are representative of increasing in the rate) (this result obtained from two independent experiments). (The frequency of cell distribution in each phase of the cell cycle was compared between the sham exposure and the exposed group by using the Mann–Whitney U test).

To evaluate the effect of ELF-EMF on the distribution of cells in different cell cycle phases, flow cytometry was performed. As shown in Fig. 4, ELF-EMF exposure led to a significant increase in the accumulation of cells at the G2/M phase (2.56%) in the U87 cell line; whilst the other two cell lines didn’t show/show negligible distribution changes in this phase. In the case of the U251 cell line, a trivial increase in the S phase was observed (2.4%) in the exposed cells compared with its sham exposure. Furthermore, in the fibroblast cell line, ELF-EMF exposure led to an insignificant increase in cell distribution at the subG1 phase (4.88%). Interestingly, in the case of U251-P53 cells, after exposure, the frequency of cells in the sub-G1 and G2/M phases increased (0.92, and 1.1% respectively), and the distribution of cells in the S phase decreased (Fig. 4, and Table2).

**Table 2:** Rate of the cells distribution in different cell cycle phases and their relation with percentage of apoptosis.

Cell line	G1-Freq%	SubG1-Freq%	S-Freq%	G2-Freq%	Apoptosis%
Fibroblast	2.66	4.88	− 8.43	0.99	2.18
U251	− 1.1	− 0.3	2.4	− 1.79	2.47
U87	− 4.6	1.43	− 0.18	2.56	11.39
U251-P53	0.28	0.92	− 1.16	1.1	8

Data represents the results obtained from the exposed cells minus the results of the sham exposure (delta).

### Real-time PCR

**Figure 5:**
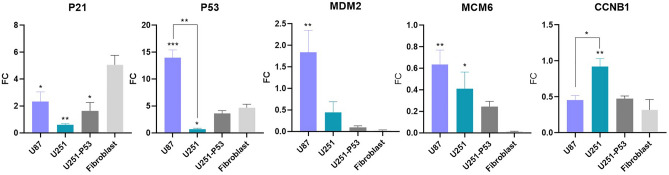
Changes in the mRNA expression level of *P53*, *P21*, *MDM2*, *MCM6*, and *CCNB1* genes in U87 and U251 cell lines after ELF-EMF exposure. The expression level of *P53*, *P21,* and *MDM2* genes increased in U87, U251-P53, and fibroblast cell lines (as data are presented as fold change (FC), the values more than 1 are considered as increasing, and values less than 1 are considered as decreasing). MCM6 and CCNB1 decreased in all three mentioned cell lines. In the case of U251, *P53*, *P21*, and *MCM6* were down-regulated after exposure. When comparing the alteration in the expression level of these genes in U87 and U251 cells, it came out that, the *P53* expression level was much lower in the U251 (*p value* = 0.0057), and *CCNB1* was much higher in U251 (*p value* = 0.0348) (this result obtained from two independent experiments and one-way ANOVA statistical analysis performed to analyze the results).

To verify the effect ELF-EMF on the important genes’ expression involved in the cell cycle progression and/or apoptosis, alteration in the expression level of five genes (*P21*, *P53*, *MDM2*, *MCM6,* and *CCNB1*) was assessed by qRT-PCR. According to the real-time PCR data, the mRNAs expression level of *P21*, *P53,* and *MDM2* were increased in the exposed group of U87 cells compared to the sham exposure (about 2.5, 14, and 1.8-fold more, respectively). While the expression level of *CCNB1* was decreased following ELF-EMF exposure in these cells. The mRNA expression level of *P21*, *P53*, and *MCM6* down-regulated in U251 cells after exposure. The expression level of *P21* and P53 increased in the U251-P53 and fibroblast cell lines after exposure (for *P21* 1.6 and fivefold more; for *P53* 3.6, and 4.65-fold more, respectively in U251-P53 and fibroblast). When comparing the expression level of the mentioned genes between these two cell lines, we realize that the expression level of *P53* increased and CCNB1 decreased much more, after exposure, in the U87 cells than in the U251 cells (Fig. 5).

### Transition electron microscopy (TEM)

**Figure 6:**
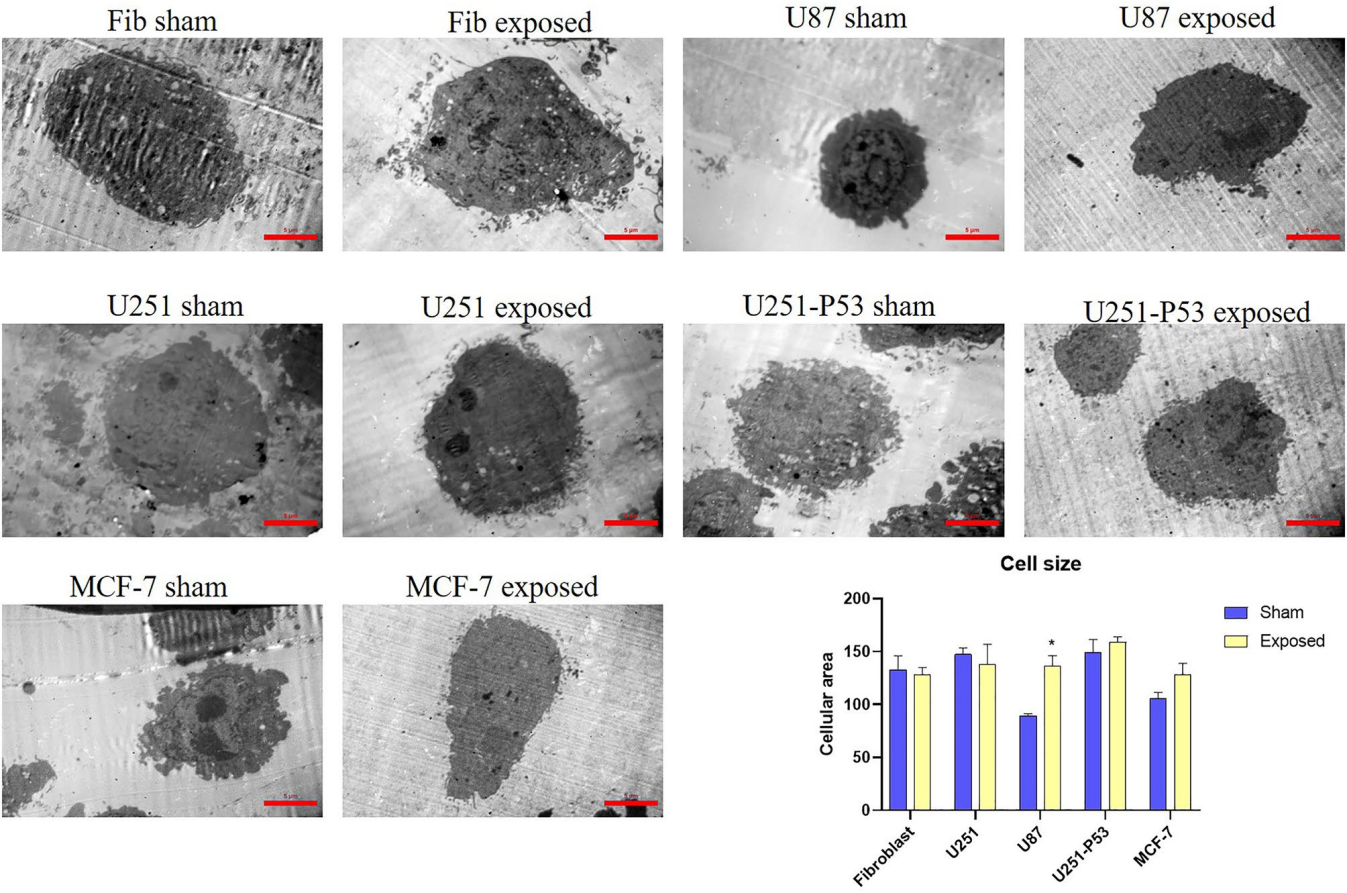
Images obtained from transmission electron microscopy to evaluate alteration in the cell size of U87, U251, U251-P53, MCF-7, and fibroblast cells after exposure to ELF-EMF. Results showed that cell size increased significantly in U87 cells (*p value* = 0.0231) compared to its sham exposure. While U251 and fibroblast cells didn’t show significant changes. U251-P53 and MCF-7 cells showed an increase in their cell size; although this increase was not statistically significant (*p value* = 0.4132, and, *p value* = 0.1271, respectively) (Mann–Whitney U test was used to compare cells’ size in between two groups (exposed and sham exposure) of each cell line).

To further assess the impact of ELF-EMF exposure on the cells’ shape and size, transmission electron microscopy (TEM) was performed. Results demonstrated slight changes in the cell size of the fibroblast and U251 cells compared with their corresponding sham exposure. This alteration was significantly more in the U87 cells (about 1.7-fold more compared with its sham exposure, *p value* = 0.0231). U251-P53 and MCF-7 cells’ size increased about 1.06, and 1.2-fold after exposure; however, this increase was not statistically significant (*p value* > 0.05) (Fig. 6).

## Discussion

### Apoptosis induction

Glioblastoma is one of the poorest prognosis cancers of primary brain tumors with a high mortality rate and poor prognosis. Current therapy options for glioblastoma include surgery, chemotherapy, and radiotherapy which are prescribed mostly as combination therapy. In this regard, the treatment of glioblastoma is a serious challenge due to the confined number of therapeutic options available^14–16^. Recently, numerous studies have shown that magnetic field (MF) therapy provides a new safe treatment method with the advantage of high efficiency and low side effects^17,18^. Low-frequency magnetic fields such as ELF-EMF are reported to have the potential to affect crucial cancer-related biological processes including apoptosis, the regulation of immunity, and suppression of angiogenesis. Moreover, amounting studies have shown that low-frequency magnetic fields could inhibit cancer proliferation and have tumor inhibitory effects; however, the mechanism of this technique remains poorly understood^19–21^. It is important to mention that ELF-EMF may interfere with some chemotherapeutic interventions; some studies have shown that it may promote the cytotoxicity and on the other hand others have shown that it may play as an inhibitory factor. It seems that the outcome in this area, at least at some steps, is due to the mechanism of action of the ELF-EMF which is related to the reactive oxygen species (ROSs) production. Moreover, these outcomes are strictly dependent to the characteristics of the fields (intensity, frequency, and time of exposure)^22–26^.

According to the previous study, electric fields such as Tumor Treating Fields (TTFields) exert an anti-cancer effect in a *P53*-dependent manner^6^. Previously, we showed that ELF-EMF has the potential to trigger apoptosis/necroptosis in different breast cancer cells^27,28^; here, by selecting two different GBM cell lines with different *P53* statuses (U87 and U251), at the first step, we tried to investigate the dependency of ELF-EMF anti-cancer effect on this gene. In the next step, the relation between this exposure, cell cycle progression, and cell size is assessed further at the molecular level. The annexin V/PI flow cytometry results revealed that ELF-EMF induces apoptosis in U87 cells much more than in U251 cells (meager apoptosis was seen in the normal fibroblasts after exposure). As we know *P53* status in U87 is wild-type and in U251 it has the mutation. This result may reflect the possible footprint of this gene in ELF-EMF-dependent apoptosis induction and different cellular response to this exposure (discussed in more detail in the next paragraphs).

### Relationship between the examined genes expression and cell cycle arrest

To investigate the impact of ELF-EMF on cell cycle progression, important gene expression in this area (*P21*, *P53*, *MDM2*, *MCM6,* and *CCNB1*) was assessed by real-time PCR. Results demonstrated that *P53*, *P21*, and *MDM2* up-regulated in U87, but they showed no significant changes in U251. On the other hand, *MCM6* and *CCNB1* were down-regulated in U251 and U87. *P21* can be directly regulated by the *P53*, and it is not out of mind to expect their expression pattern to act similarly; moreover, *P21* can induce apoptosis and cell cycle arrest in the G1/S and G2/M phase of the cell cycle^29,30^ (these are following the data obtained from annexin V/PI and cell cycle flow cytometry). *MDM2* (mouse double minute 2) is a negative regulator of *P53*; *MDM2* regulates the *P53* level and its activity^31,32^. Our results revealed that the *MDM2* expression level increased in the U87 cell line; although we expect the latter increase to exert a negative effect on the *P53* expression/function, as the expression level of *P21* increased, we may conclude that *MDM2* didn’t succeed to exert its negative regulation on the *P53*. This may be at least in some steps, due to the increase in *MDM2* was not enough to overcome and block the *P53* increase (*P53* expression level increased much more in the U87 cells compared with *MDM2* increasing).

In the case of U251, the expression level of *CCNB1* and *MCM6* decreased in this cell line after exposure. *MCM6* is a member of the mini-chromosome maintenance family (MCM), which plays an important role in limiting the replication in each cell cycle^33^. *MCM6* has helicase activity which leads to the recruitment of DNA polymerase and the initiation of DNA replication^34^. MCM6 is a candidate marker for cell proliferation; an increase in *MCM6* levels indicates the proliferation of malignant cells. Overexpression of *MCM6* is found in many human cancers, and knocking down it significantly inhibits cell proliferation^35–39^. From a cell cycle point of view, inhibiting *MCM6* can delay the progression of the cell cycle from G1/S by down-regulating the expression of cell cycle checkpoint proteins. This result is following cell cycle flow cytometry data for U251, where a meager increase in the population of cells in the S phase is revealed (Fig. 4B). *CCNB1*, encoded by the CCNB1 gene, belongs to the cyclin super family ^40^. CCNB1 has a key role in Cdk1 regulation and transition from the G2 phase to mitosis progression^41^. *CCNB1* also called cyclin B1, has shown that is closely associated with tumor progression and was highly expressed in tumor tissues or cells^42^. Our results demonstrated that down-regulating of *CCNB1* is more significant in U87 cells than U251; hence, it is not out of mind to expect that the rate of cells in the G2/M phase of the cell cycle shall be more in the exposed U87 cells compared with the U251. Along with the crucial role of this gene in the transition of the cell cycle from G2/M, *CCNB1* down-regulation can also up-regulate *P53* and subsequently *P21* which in turn leads to cell cycle arrest^43^. To confirm this, it is important to mention that, Zhang et al. and Li et al. showed that down-regulating *CCNB1* prevents cancer progression through *P53* signaling pathway activation^41^ which has a key role in mediating the responses of cancer treatments^44^. Table 3 summarizes the role of the mentioned genes in the progression or inhibition of the cell cycle (Table 3).

**Table 3:** Role of P21, P53, MDM2, MCM6, and CCNB1 role in cell cycle arrest/progression.

Genes	Cell cycle arrest	Progression cell cycle
P21	G1/S, G2/M	–
P53	G1/S, G2/M	–
MDM2	–	Negative control of p53
MCM6	Downregulation results in delay in S/G2	G1/S
CCNB1	Downregulation results in G2/M arrest	G2/M

### P53 status

*TP53* is a major tumor suppressor ^45^ that has a central role in various cellular responses including apoptosis ^46^, DNA repair ^47^, and cell cycle ^48^. Amounting of recent studies have shown that not only does TP53 serve a central role in the regulatory network of tumorigenesis, but also its status is closely associated with the disease progression and survival of patients with GBM during radio/chemo therapy^49,50^; this gene is also considered as a targeted therapy for cancer treatment^6^. The importance of *P53* in the response to the treatment in GBM patients, on one hand, and on the other hand possible footprint of *P53*-dependent apoptosis induction by the electrical field^6^; by choosing two GBM cell lines with different *P53* status (U87 as wild type and U251 as mutated type (Table 4)), we struggled to assess the possible role of this gene status in the response obtained after ELF-EMF exposure. Apoptosis may be induced by *P53*-dependent or independent pathways. Our results answered some parts of this question, as we can see, the apoptosis rate induced in the U87 cells after ELF-EMF exposure is much more than in the U251 cells. It is shown that overexpression of *P53* in the U87 cell line after exposure may be the main reason for apoptosis induction by radiation^51,52^, as in the U251 cell line, *P53* is mutated it is not out mind to expect that the underlying and *P53*-dependent pathways which may result in apoptosis after treatment, do not function correctly^13^. The results obtained for the MCF-7 (with wild type P53) or U251-P53 (U251 cells that received vector overexpressing *P53*) also confirmed the role of this gene in the apoptosis induction by ELF-EMF in GBM cells. As we can see, in MCF-7 cells or after overexpression of P53 in U251 cells, the apoptosis rate increased in the U251-P53 cells.

**Table 4:** P53 status in U87 and U251. U87 cell line has wild-type p53, however U251 has the mutated form of this gene.

Cell lines	P53 status	Mutation information				
		In protein	In cDNA	Exon	Chromosome position	SNIP RS
U87	WT	–	–	–	–	–
U251	MT	p.Arg273Leu	c.818G>T	exon 8 of 11 position 36 of 137 (coding)	17p13.1 (7673802)	Rs28934576

We can see the mutated amino acid location in the protein from protein structure obtained from the PDB.
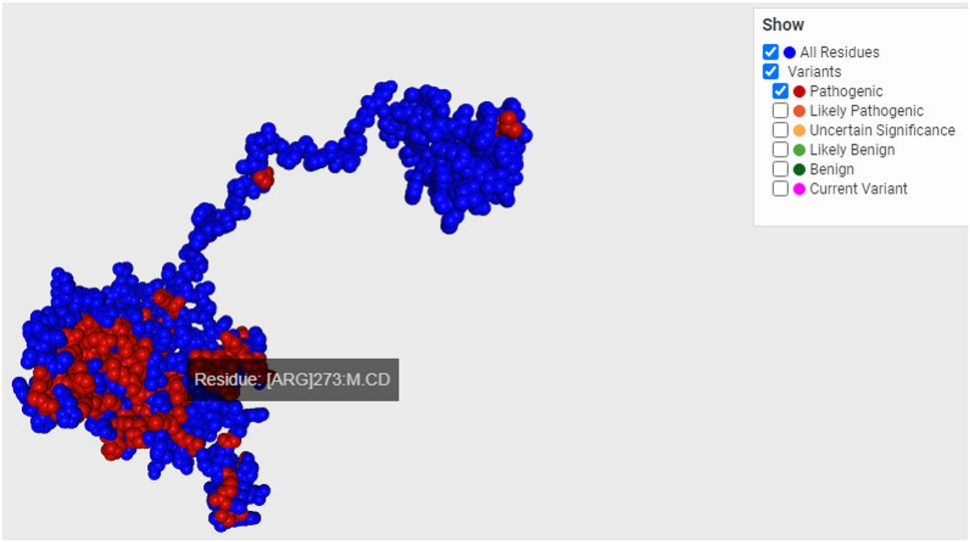

### Relationship between G2/M cell cycle arrest and cell size

Our results revealed that after exposure to ELF-EMF, the rate of U87 cells in the G2/M phase of the cell cycle increased (as described earlier can be due to the up-regulation of *P53* and subsequently *P21* in these cells). As we know during the interphase of the cell cycle (G1, S, and G2) cell growth occurs and human cells become tetraploid after the replication in the S phase. Hence, it is not out of mind to say that before the mitosis and cell division (karyokinesis and cytokinesis) the volume of cells is more than those which reside in the early phases of the cell cycle (specifically before the S phase). One of the main mechanisms which have been proposed for tumor treating field (TTF) to act as an anti-cancer modality, is through affecting the microtubules and actin dynamics. With this point of view, TTFields interfere with the normal formation of the spindle in cell division and arrest cells in the metaphase of mitosis, which can be reflected as G2/M arrest in the cell cycle flow cytometry^53^. This phenomenon increases the cell size of the treated cells. In addition to G2/M arrest, nuclear size can increase due to cellular stresses such as that caused by reactive oxygen species, senescence, and necrosis ^54^. The results obtained from the TEM here revealed that the cell size of the U87 cells, which had G2/M arrest, increased significantly after exposure to the ELF-EMF; the underlying mechanism of action here seems to be the same as the TTField.

## Conclusion

ELF-EMF anti-cancer effect is sensitive to the *P53* status in the exposed cells (U87 cells with wild-type *P53* are more sensitive to this field compared with the U251 which has a mutated form of *P53*). Our results suggest that G2/M arrest and increase in the cell size can be the main mechanism of apoptosis induction by these fields. These three (*P53* status, G2/M arrest, and increase in the cell size) are similar between the ELF-EMF and renowned TTFields, which can be a lantern that helps in reaching ELF-EMF to the bedside, just like its cousin the TTField.

## Data Availability

Data will be available on request to the corresponding author (Mohammad Amin Javidi; email: Javidi@acecr.ac.ir).
